# Examining Cash Expenditures and Associated HIV-Related Behaviors Using Financial Diaries in Women Employed by Sex Work in Rural Uganda: Findings from the Kyaterekera Study

**DOI:** 10.3390/ijerph20095612

**Published:** 2023-04-23

**Authors:** Larissa Jennings Mayo-Wilson, Summer K. Peterson, Joshua Kiyingi, Proscovia Nabunya, Ozge Sensoy Bahar, Lyla S. Yang, Susan S. Witte, Fred M. Ssewamala

**Affiliations:** 1 Department of Health Behavior, Department of Maternal and Child Health, Gillings School of Global Public Health, University of North Carolina, 135 Dauer Drive, Chapel Hill, NC 27599, USA; 2 Brown School, Washington University in St. Louis, One Brookings Drive, St. Louis, MO 63130, USA; 3 International Center for Child Health and Development, Masaka Field Office, Mutuba Ave, Masaka 319, Uganda; 4 School of Social Work, Columbia University, 1255 Amsterdam Avenue, New York, NY 10027, USA

**Keywords:** HIV, sexual risk behaviors, financial diaries, female sex workers, Uganda, clinical trial

## Abstract

Background: Women employed by sex work (WESW) have a high risk of human immunodeficiency virus (HIV) infection and experience economic barriers in accessing care. However, few studies have described their financial lives and the relationship between expenditures and HIV-related behaviors. Methods: This exploratory study used financial diaries to collect expenditure and income data from WESW in Uganda over 6 months. Data were collected as part of a larger trial that tested the efficacy of an HIV prevention intervention method. Descriptive statistics were used to quantify women’s income, relative expenditures, and negative cash balances. Bivariate and multivariate logistic regressions were used to examine the odds of sexual risk behavior or use of HIV medications for several cash scenarios. Results: A total of 163 WESW were enrolled; the participants mean age was 32 years old. Sex work was the sole source of employment for most WESW (99%); their average monthly income was $62.32. Food accounted for the highest proportion of spending (44%) followed by sex work (20%) and housing expenditures (11%). WESW spent the least on health care (5%). Expenditures accounted for a large but variable proportion of these women’s income (56% to 101%). Most WESW (74%) experienced a negative cash balance. Some also reported high sex work (28%), health care (24%), and education (28%) costs. The prevalence of condomless sex (77%) and sex with drugs/alcohol (70%) was high compared to use of ART/PrEP (Antiretroviral therapy/Pre-exposure prophylaxis) medications (45%). Women’s cash expenditures were not statistically significantly associated with HIV-related behaviors. However, the exploratory study observed a consistent null trend of lower odds of condomless sex (adjusted odds ratio (AOR) = 0.70, 95% confidence interval (CI): 0.28–1.70), sex with drugs/alcohol (AOR = 0.93, 95% CI: 0.42–2.05), and use of ART/PrEP (AOR = 0.80, 95% CI: 0.39–1.67) among women who experienced a negative cash balance versus those who did not. Similar trends were observed for other cash scenarios. Conclusion: Financial diaries are a feasible tool to assess the economic lives of vulnerable women. Despite having paid work, most WESW encountered a myriad of financial challenges with limited spending on HIV prevention. Financial protections and additional income-generating activities may improve their status. More robust research is needed to understand the potentially complex relationship between income, expenditures, and HIV risk among vulnerable sex workers.

## 1. Introduction

Sex work has historically been recognized as a driver of HIV incidence; women employed by sex work (WESW) continue to have heightened risk and adverse impact from HIV [1,2]. According to the Joint United Nations Programme on HIV and AIDS (UNAIDS), HIV incidence among people who are employed by sex work is 21 times higher than HIV incidence among the general population of adults, aged 15–49, who do not exchange sex for money [1]. WESW account for 39% of all new HIV infections in sub-Saharan Africa, most proximally due to inconsistent condom use in the absence of anti-retroviral medications [1,3].

The decision for many women regarding whether and how to engage in sex work is motivated by financial considerations [4,5,6,7,8]. Studies have shown that to pay for living expenses, sex workers experiencing economic insecurity often rely on higher-paying but riskier sexual transactions, such as condomless sex and anal sex, which increase the likelihood of HIV transmission [9,10,11,12,13]. Riskier unprotected sex is associated with up to 43% higher client-paid fees [9,10,11,12,13]. Women experiencing financial distress, including food insecurity, debt, and lower earned income, are also more likely to have multiple sex partners and engage in unsafe sexual practices, such as sex with partners of unknown HIV status and sex while high or drunk [14,15,16,17]. One explanation is that economically insecure women are less able to negotiate condom use or substance-free sex with higher-earning sexual partners [18,19]. They are also not empowered to inquire about HIV status [14]. Another explanation suggests that the psychological effects of economic insecurity (e.g., depression, anxiety) diminish motivations to protect against HIV [20,21,22,23], thus leading to engagement in unsafe sexual practices. In addition, uptake of HIV care-seeking behaviors, such as testing or initiation of antiretroviral medications, can be impacted by economic barriers [24,25,26,27]. For example, clinical fees and transportation costs to test for HIV and attend medical appointments can be prohibitive for financially insecure women [24,25,26,27,28]. There are also monetary disincentives associated with losing time away from paid work (i.e., lost wages) to attend medical appointments [26,29,30]. Even when HIV services are free, research has shown that women with limited or no cash savings are less likely to test for HIV due to expected future expenses [27,30]. 

However, little is known about the financial lives of WESW. Economic-strengthening interventions (ESI), such as savings groups, cash transfers, and income-generating activities, aim to modify more distal economic factors associated with HIV by increasing economic security to reduce women’s financially-driven decisions about sex partners, sexual practices, and sexual health care-seeking behaviors [31,32,33,34]. These interventions are praised for doing more than centering on sexual behaviors [4]. However, there is a dearth of information about how WESWs cash inflows and outflows impact uptake of sexual and care-seeking behaviors related to HIV [35,36,37]. This limits our understanding of the mechanisms of change in ESIs. Collecting data on the financial transactions of WESW could better inform the design of ESIs for HIV resilience and risk reduction [35,36,37].

To fill this gap, we used financial diaries to understand the economic lives of WESW and examine associations between women’s finances and their sexual and HIV care-seeking behaviors. Financial diaries are a high-frequency data collection methodology used to obtain detailed quantitative accounts of financial transactions to document fluctuations in earnings and expenditures [38,39,40,41]. Using paper or tablets, they are most commonly used in resource-poor settings to characterize episodic poverty among individuals with low literacy and numeracy skills [4,35,36,42]. Financial diary participants are asked to note all cash inflow and outflow transactions for each day, including the date, expense amount, and expense description [38,39,40,41]. In some cases, bartering and in-kind exchanges are also documented [38,41]. Although the literature is scant, in recent years financial diary studies among WESW have shown that diaries are an acceptable method for examining transactional sex and women-controlled finances (not household finances) [37,42] and that a substantial proportion of women’s expenses directly support sex work (e.g., clothing, alcohol, beauty products) [4,35,36]. Financial diary studies have also shown that cash expenses represent most of WESWs spending, income is erratic from week to week, and savings are regularly withdrawn [4,35,36,42]. However, to our knowledge, no financial diary study among WESW has examined the association between participants’ finances and their engagement in sexual risk and care-seeking behaviors. This study is also among the first and largest financial diary studies among WESW to be implemented in Uganda. The two main objectives were: (1) to describe the financial lives of WESW who are experiencing economic barriers to care and for whom risk of HIV infection is high; and (2) to examine the relationship between cash expenditures and HIV-related sexual and care-seeking behaviors.

## 2. Methods

### 2.1. Study Design

Data were collected as part of a larger 24-month multisite, longitudinal clinical trial (the Kyaterekera Project) that tested the efficacy of adding savings, financial literacy, and mentorship to traditional HIV risk reduction education on reducing incidence of HIV and other sexually transmitted infections (STIs) among a cohort of WESW in the greater Masaka region in southern Uganda [43]. In the larger clinical trial, data collection was conducted using six core methods: (1) process documentation tools; (2) computer-assisted quantitative surveys; (3) biological assays; (4) medical chart reviews; (5) qualitative in-depth interviews; and (6) monthly financial diaries. The current study evaluates data taken primarily from the monthly financial diaries (FD) and quantitative surveys.

### 2.2. Setting

There are an estimated 1895 registered WESW within targeted “hotspots” or high HIV prevalence areas of the study region. Most WESW (77%) in the region reported a new STI diagnosis in the prior 12 months [44]. HIV prevalence among Ugandan WESW is 61%, over 10 times higher than HIV prevalence in Ugandan adults who are not employed by sex work (5.8%) [45,46,47,48]. 

### 2.3. Participants

WESW enrolled in the larger clinical trial were recruited from October 2019 to February 2020 using community and peer liaisons from the International Center for Child Health and Development (ICHAD). A detailed description of the larger trial’s methodology, including use of computer-assisted quantitative surveys, w described in a previously published protocol manuscript [43]. In sum, women were eligible to participate if they were: employed by sex work; aged 18 years or older; reported having engaged in vaginal or anal intercourse in the past 30 days in exchange for money, alcohol, or other goods; and reported having had at least one episode of unprotected sexual intercourse in the past 30 days with either a paying, non-paying, casual, or regular sexual partner. A total of 542 WESW were recruited and enrolled into the larger clinical trial and randomized to experimental (N = 356) versus control (N = 186). However, only 163 women of the 356 assigned to the experimental group were able to complete financial diary booklets (and, therefore, enrolled in the FD sub-study) due to interruptions to study activities from the COVID-19 pandemic.

### 2.4. Financial Diaries Data Collection

**Figure 1:**
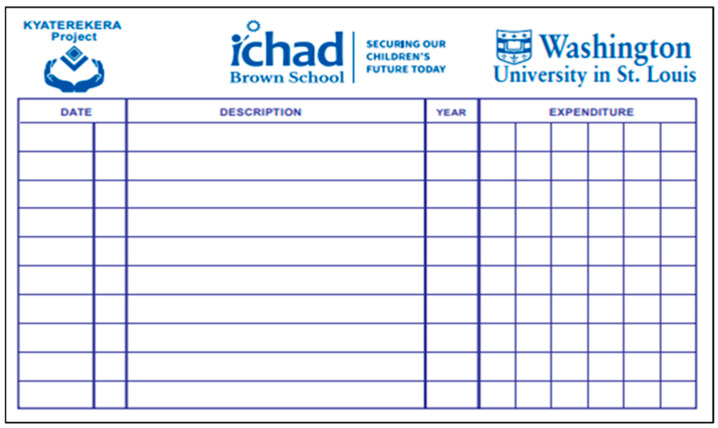
Example page of financial diaries booklet used to measure cash expenditures and income among women employed by sex work (WESW) over the 6-month data collection period.

The study collected FD data for 6 months from October 2019 to March 2020. A description of the FD methodology used by the Kyaterekera Project is under review for publication elsewhere [49]. In sum, the FDs used in the study were paper-based booklets adapted from previously published FD methodologies [36,39,40,41,42]. English and Swahili language versions of the booklets were available for use. Each FD booklet included several pages sufficient for one month’s duration with tabular entries for date (e.g., day, month, year), descriptions of items bought, and the amount spent in Ugandan Shillings (Figure 1). 

WESW were provided one writing pen and trained for ~2 h on how to record their expenditures using the FD. WESW with limited literacy skills were encouraged to have a trusted friend or relative complete their diaries. Specifically, WESW were asked to record all cash expenditures, representing purchased goods or services using the FD. They were also asked to record all non-sex work income, such as cash deposits, gifts, study-administered matched savings, or other monies. Given the sensitivity of sex work in Uganda and the need to protect their privacy, WESW were not asked to record income received from sex work. They also were not asked to document non-cash transactions, such as in-kind and barter transactions. Instead, monthly sex work income data were obtained using a more confidential, computer-assisted survey and calculated as the average of monthly sex work income reported at study baseline and follow-up. In addition, women’s names, housing addresses, and other personal information were excluded from the FD to protect privacy. 

To enhance FD data quality, each WESW had a one-on-one monthly meeting with a study team member to review her diary, assess it for completeness, and clarify any unclear or inconsistent entries. The study team member then collected each WESWs paper-based FD for the month and provided a new booklet for the next month’s duration. All FD data were then extracted by study ID and entered into a larger study database. During national travel restrictions due to COVID-19, quality meetings for FDs with WESW were conducted by phone and study members collected FD booklets once travel restrictions were removed. WESW did not receive remuneration for completing the FDs, nor were there any penalties for incomplete, lost, or missing diary entries. 

### 2.5. Quantitative Survey Data Collection

All WESW additionally completed a computer-assisted quantitative survey at the time of study enrollment that included sections on demographic information, household characteristics, sex work characteristics, economic measures, sexual risk behaviors, and uptake of antiretroviral medications. Survey questions were provided in English or Swahili and included closed-ended response prompts. WESW were invited to respond to survey questions following administration of informed consent. We used sexual behavioral and care-seeking data from the quantitative surveys to assess the association between WESWs cash expenditures and HIV-related behaviors.

### 2.6. Analysis

All data were analyzed using STATA BE, Version 17 (Stata Corporation, College Station, TX, USA). Firstly, we used descriptive statistics to assess baseline demographic characteristics of WESW enrolled in the FD sub-study relating to age, education, partnership status, household size, savings, debt, number of paying clients, and non-sex work employment. Two sample *t-*tests and chi-square statistics were then used to examine differences in demographic characteristics between WESW enrolled in the FD sub-study versus WESW enrolled in the larger clinical trial (e.g., parent study). Secondly, we converted all FD data from Ugandan Shillings (UGX) to US dollars (USD) using the average exchange rate of 3794.35 UGX per 1.00 USD [50]. Total cash expenditures for the study sample were then calculated by month based on the sum of all expenditures for all women in a given month. We also calculated mean cash expenditures by month based on the average of all cash expenditures of each woman in a given month. Using item descriptions in the FD booklets, we then labeled each expenditure by one of 15 detailed codes (e.g., lodging, food, clothing, cosmetics, medications, leisure, etc.) to understand the distribution of types of expenses among WESW. For ease of interpretation, these detailed codes were then collapsed into 6 expense categories relating to food, housing, sex work, health, education, or other living expenses (e.g., toiletries, leisure, etc.). Total and mean cash expenditures by month were then calculated for each of the 6 expense categories, including the percentage of all cash expenditures attributable to a particular expense category. 

Next, we estimated each woman’s total monthly cash income based on the sum of non-sex work income reported in the FDs and sex work income reported in the computer-assisted quantitative survey. These estimates were then used to calculate the total and mean cash income by month for the sub-study sample. To examine experiences of income insecurity among WESW, we quantified the number of WESW with a negative cash balance each month, defined as total cash expenditures exceeding total cash income in a given month. We then summed the total negative cash balance each month and calculated the average negative cash balance each month among women with a negative cash balance in the given month. We also calculated the number of women with exceedingly high sex work costs, health care costs, or education costs, defined as having monthly categorical expenses exceeding the average categorical expenses of the sub-study sample. These three high costs (e.g., sex work, health care, and education) in addition to experiencing a negative cash balance comprised four cash expenditure scenarios that were further explored. 

As a final step, bivariate and multivariate logistic regressions were used to examine the relationship between women’s cash expenditures and HIV-related behaviors. We calculated the odds of engaging in condomless sex in the last 90 days, sex with alcohol or drugs in the last 90 days, and uptake of antiretroviral medications (ART/PrEP) at the time of study enrollment for each cash expenditure scenario; the scenarios represented WESW who had a negative cash balance, high sex work costs, high health care costs, or high education costs, respectively, versus those who did not. We controlled for age and education in adjusted regressions. Given the small sample size, these regressions were used only to explore preliminary associations. All analyses were considered statistically significant at *p* < 0.05 or when the 95% confidence interval (CI) did not include the null odds ratio of 1.0. 

## 3. Results

### 3.1. Participant Characteristics

Table 1 describes the sample’s demographic characteristics. A total of 163 WESW were enrolled in the financial diaries (FD) sub-study (Table 1). The mean age was 32.2 years, ranging from 18 to 55. Most WESW had less than primary education (59.5%), were single/unpartnered (78.5%), and lived in households with only one adult (57.1%). Overall financial status was low to moderate. Sex work was the sole source of employment for nearly all WESW (98.8%) with 48.5% of WESW having savings compared to 73.0% having debt. There were no significant differences in demographic characteristics between WESW enrolled in the FD sub-study versus those who were not.

### 3.2. Cash Income and Expenditures

**Figure 2:**
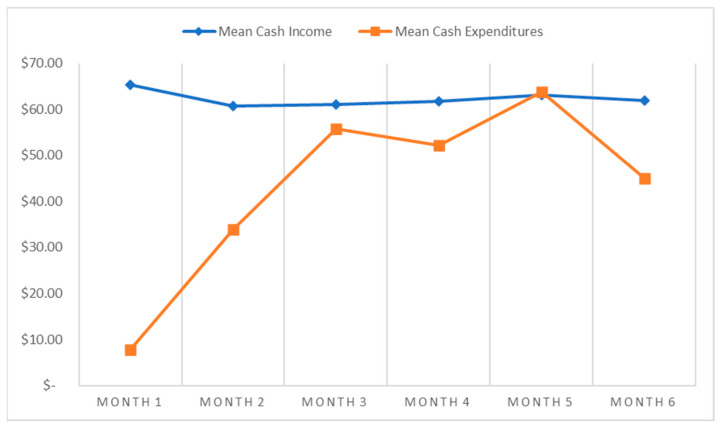
Mean cash income versus mean cash expenditures by month in USD for women employed by sex work (WESW) over the 6-month financial diaries data collection window (N = 163).

Table 2 and Table 3 respectively describe total and mean cash income and expenditures by month and across the full 6-month data collection period. Reported cash income was relatively consistent over time. The sub-study sample of WESW reported $60,948 USD in total cash income over the 6-month data collection period (Table 2), ranging from $9990 USD to $10,636 USD in a given month. The average monthly cash income per woman ranged from $60.71 to $65.25 USD (Table 3). WESWs reported average monthly cash expenditures were more variable over time with average expenditures being lowest at the start of data collection ($7.77 USD, Month 1 and $33.84 USD, Month 2) and highest at the peak of data collection ($52.20 USD, Month 4, $63.82 USD, Month 5) (Table 3). Food expenditures accounted for the highest proportion of spending (44%) followed by sex work expenditures (20%) and housing expenditures (11%) (Table 2). Education (10%) and health care (5%) expenditures accounted for the smallest proportions. Mean monthly cash expenditures accounted for a large but variable proportion of mean monthly income (55.7% to 101.1%) (Table 3). The total sample of WESW had a mean monthly cash income that was greater than the sample’s mean monthly cash expenditure, representing no negative cash balance at the level of the study sample (Figure 2). However, 120 WESW (73.6%), ranging from 7 to 67 WESW in a given month, had a negative cash balance (Table 3). Among women experiencing a negative cash balance, the mean monthly deficit ranged from −$56.07 USD to −$94.80 USD (Table 3). A small proportion of WESW also reported above-average sex work costs (28.2%), health care costs (23.9%), and education costs (27.6%) as compared to their peers (Table 4).

### 3.3. HIV-Related Behaviors

The prevalence of sexual risk behaviors was high in the sub-study sample (Table 4). Most WESW reported having one or more acts of condomless sex in the last 90 days (77.3%, n = 126) or one or more acts of sex under the influence of drugs and/or alcohol in the past 90 days (69.9%, n = 114). Less than half (45.4%, n = 74) of WESW had initiated an HIV antiretroviral medication (ART/PrEP) at the time of study enrollment (Table 4). 

### 3.4. Association of Cash Expenditures and HIV-Related Behaviors

Table 5 depicts the proportion of WESW reporting sexual risk behaviors or uptake of antiretroviral medications (ART/PrEP) for each of the four cash expenditure scenarios relating to deficit spending or above-average spending. Table 6 shows the crude and adjusted odds ratios of sexual risk behaviors or uptake of ART/PrEP by cash expenditure scenario. On the whole, WESWs cash expenditures were not statistically significantly associated with HIV-related behaviors. However, there was a consistent trend of WESW who experienced a negative cash balance being less likely to engage in condomless sex (AOR = 0.70, 95%CI: 0.28–1.70), less likely to engage in sex with alcohol or drugs (AOR = 0.93, 95%CI: 0.42–2.05), and less likely to initiate ART/PrEP (AOR = 0.80, 95%CI: 0.39–1.67) as compared to women who had not experienced a negative cash balance (Table 6). There was also a consistent though insignificant trend of WESW who reported high sex work costs being less likely to engage in condomless sex (AOR = 0.86, 95%CI: 0.38–1.96), less likely to engage in sex with alcohol or drugs (AOR = 0.76, 95%CI: 0.37–1.59), and less likely to initiate ART/PrEP (AOR = 0.99, 95%CI: 0.49–2.03) as compared to WESW who reported below-average sex work costs (Table 6). Similar findings were observed in all unadjusted analyses and in adjusted analyses relating to health care and education costs (Table 6).

## 4. Discussion

This study aimed to describe the financial lives of women employed by sex work (WESW) and examine the relationship between their cash expenditures and HIV-related behaviors. Using monthly financial diaries, we found that despite having paid work, the economic lives of WESW entailed low and insufficient income with fluctuating high expenses. The financial diaries revealed many of the expected financial challenges of cash-poor populations. For most WESW, income was largely spent on housing and food with little surplus for expenses not related to basic needs. Monthly expenditures also consistently accounted for a large proportion of monthly income, often exceeding the amount of cash that WESW had on hand. As a result, there was a high prevalence of negative cash balances. Nearly three-fourths of WESW had expenses exceeding their income, which likely contributed to the reported high debt and low savings rate in the sample. The financial diaries also showed that WESW had the lowest spending on health care. There were few transactions relating to prevention of HIV and other sexually transmitted infections. Our findings of negative cash flow, fluctuating expenses, high debt, and low spending on health care corroborate findings from other studies [4,35,36]. This is concerning because WESW have a high risk of HIV infection and report experiencing economic barriers to care.

The similarities of our results compared to other financial diary studies among WESW underscore the importance of developing economic interventions that provide greater financial protections and income security to WESW. This may include income supplements, emergency cash assistance, debt relief, savings plans, or training in budgeting and financial management. In particular, economic interventions that can be applied towards health care costs may reduce economic barriers to condom use, testing, and uptake of HIV medications. Equally important, our findings suggest that for some WESW identification of non-sex work employment may help to reduce cash deficits by supplementing income when expenses are high. However, this may be difficult to undertake in settings where women have few employment options with comparable pay. Our study found that WESWs mean monthly income was $62.32 USD, comparable to the Gross National Income (GNI) per capita monthly income for adults in Uganda of $61.70 USD [51]. Two previous financial diary studies among WESW in Cote D’Ivoire and Ethiopia also found that WESW earned incomes equal to or higher than the per capita income [4,36]. These findings suggest that although the amount of pay from sex work was insufficient for many WESW, it is comparable to the national living wage. A range of innovative efforts will likely be needed to identify paid work for WESW that matches or exceeds sex work income in some settings. 

The monthly financial diaries also showed that a large proportion of women’s sex work income is allocated back to sex work business costs. In fact, sex work expenditures made up the second largest expenditure category (~20%) among WESW in our study. This is similar to findings from other financial diary studies among WESW that observed that roughly 10 to 30% of financial diary expenditures were for sex work expenses (e.g., supplies, cosmetics, rented client venues) [4,36]. Therefore, economic interventions that offset sex work business costs, such as by purchasing supplies or subsidizing sex work venues, may enable WESW to avoid negative cash balances and invest in other health and social goals.

A final implication relates to the association between cash expenditures and HIV-related behaviors. While the prevalence of sexual risk behaviors in the study sample was high with lower use of ART/PrEP, we found no statistically significant association with the expenditures reported in financial diaries. However, we did observe a trend across all null analyses of lower odds of sexual risk behaviors and use of ART/PrEP among WESW who were experiencing financial insecurity. For example, WESW with a negative cash balance or high monthly costs were less likely to engage in sexual risk behaviors. This unexpected finding may be explained by lower earnings among sex workers who require clients to use condoms. It may also mean that WESW with higher costs also earn higher incomes and are better positioned to negotiate safe sex. WESW with higher health care costs may also be more motivated to engage in sexually protective behaviors. On the other hand, WESW experiencing high costs were also less likely to initiate ART/PrEP, which could reflect their having limited surplus money for HIV medications and its related costs (e.g., travel to the clinic, extra meals, lost wages). Ultimately, our study found that implementing financial diaries to measure WESWs financial transactions was feasible, but that more research is needed to examine the potentially complex and multidirectional influence of income, expenditures, and HIV-related behaviors.

### Limitations

The limitations of this study should be considered. Firstly, this study was exploratory and not specifically designed to assess quantitative correlations between cash expenditures and HIV risk. A larger, more robust quantitative study may identify significant associations that our study’s relatively small sample size could not detect. Secondly, the financial diaries did not measure income from sex work in real time; this was reported as a monthly estimate. It is possible that fluctuations in sex work income from month-to-month were missed. In addition, sexual behaviors and uptake of HIV medications were not measured with the same frequency as the financial diaries data which reduced the comparability of these data. Thirdly, although WESW were encouraged to document financial transactions in real time, it is conceivable that some income or expenditures were omitted or misremembered. Lastly, with the exception of age and education, this study did not account for non-economic factors that may have influenced WESWs behaviors. Despite these limitations, our study’s similarity with findings from previous research is encouraging. Our results additionally point to how future research examining intersections between financial and sexual behavioral risks might be designed.

## 5. Conclusions

This small, exploratory study found that despite having paid work, WESW encountered low earnings, negative cash balances, fluctuating high costs, and limited spending on health services. Economic-strengthening interventions for WESW are urgently needed. In addition, while these financial experiences were not statistically associated with HIV-related behaviors, we observed a consistent null trend of lower odds of sexual risk behavior and lower odds of HIV medication use among financially insecure WESW. More robust research is needed to understand the potentially complex relationship between income, expenditures, and HIV risk among vulnerable sex workers.

## Figures and Tables

**Table 1:** Demographic and sexual behavior characteristics of women employed by sex work (WESW) at time of enrollment in financial diaries sub-study (N = 163) versus parent study (N = 542).

	Sub-Study		Non-Sub-Study		*p*	Parent Study	
Demographic and sexual behavior characteristics	N	(n/N) %	N	(n/N) %		N	(n/N) %
Total sample	163	100%	379	100%		542	100%
Mean age in years (±SD)	32.2 (±7.8)	-	31.0 (±6.9)	-	0.18	31.4 (±7.2)	-
Age range in years	18–55	-	18–53	-	-	18–55	-
Age in years 18–29 ≥30	63 100	38.7% 61.3%	169 210	44.6% 55.4%	0.20	232 310	42.8% 57.2%
Highest level of education Less than primary education Primary school or more	97 66	59.5% 40.5%	247 132	65.2% 34.8%	0.21	344 198	63.5% 36.5%
Partnership status Single/unpartnered ^a^ Married/partnered ^b^	128 35	78.5% 21.5%	275 104	72.6% 27.4%	0.15	403 139	74.4% 25.6%
Adult household size ^c^ One adult Two or more adults	93 70	57.1% 42.9%	224 155	59.1% 40.9%	0.66	317 225	58.5% 41.5%
Highest household income earner ^d^ Yes No	42 28	60.0% 40.0%	86 69	55.5% 44.5%	0.53	128 97	56.9% 43.1%
Has savings Yes No	79 84	48.5% 51.5%	181 198	47.8% 52.2%	0.88	260 282	48.0% 52.0%
Has debt Yes No	119 44	73.0% 27.0%	269 110	71.0% 29.0%	0.63	388 154	71.6% 28.4%
Mean number of paying clients in the past 30 days (±SD)	26.6 (±36.3)	-	36.4 (±51.2)	-	0.54	31.3 (±47.1)	-
Has other non-sex work employment in last 12 months Yes No	2 161	1.2% 98.8%	6 373	1.6% 98.4%	0.75	8 534	1.5% 98.5%

^a^ Not partnered includes separated, divorced, widowed, and single/never married; ^b^ partnered includes legal marriage, common law marriage, and committed non-marital relationship; ^c^ ranges from 1 to 18 and includes enrolled WESW as one adult; ^d^ includes only women with adult household size >1.

**Table 2:** Total cash income and cash expenditures in USD by month and by category for women employed by sex work (WESW) who were enrolled in the financial diaries sub-study (N = 163).

	Month 1	Month 2	Month 3	Month 4	Month 5	Month 6	All Months	% of All Cash Expenditures
Total cash income ^a^	$10,636.48	$9896.30	$9962.72	$10,064.71	$10,288.60	$10,099.50	$60,948.31	-
Total cash expenditures	$1265.75	$5516.50	$9081.91	$8509.34	$10,402.34	$7328.98	$42,104.82	100%
% Total cash income	11.9%	55.7%	91.2%	84.5%	101.1%	72.6%	69.1%	-
Expense category								
Food expenses	$732.59	$2167.73	$4002.98	$3572.76	$4434.17	$3435.06	$18,345.28	44%
Sex work expenditures	$169.46	$1211.38	$1898.35	$1556.05	$2173.37	$1352.64	$8361.26	20%
Housing expenditures	$75.64	$452.25	$1015.85	$878.68	$1043.66	$1042.21	$4508.29	11%
Other living expenditures ^b^	$184.67	$992.42	$1149.63	$690.71	$999.83	$638.24	$4655.51	11%
Education expenditures	$67.34	$454.70	$392.35	$1303.18	$1381.71	$589.14	$4188.42	10%
Health care expenditures	$36.05	$238.01	$622.74	$ 507.97	$369.60	$271.69	$2046.07	5%
Number of women with negative cash balance ^c^	7	30	49	48	67	54	120	-
Total negative cash Balance ^d^	−$663.62	−$2662.41	−$4635.19	−$3905.81	−$4876.89	−$3027.64	−$19,771.55	-

^a^ Sum of individual income as reported in monthly financial diaries and computer-assisted surveys; ^b^ includes miscellaneous expenses, such as toiletries, leisure, cookware, etc.; ^c^ defined as cash expenditures > cash income; ^d^ includes only denominator of women with a negative cash balance for the given month.

**Table 3:** Mean cash income and cash expenditures in USD by month and by category for women employed by sex work (WESW) who were enrolled in the financial diaries sub-study (N = 163).

	Month 1	Month 2	Month 3	Month 4	Month 5	Month 6	All Months	% All Cash Expenditures
Mean cash income ^a^	$65.25	$60.71	$61.12	$61.75	$63.12	$61.96	$62.32 ^b^	-
Mean cash expenditures	$7.77	$33.84	$55.72	$52.20	$63.82	$44.96	$43.05 ^b^	100%
% Monthly mean cash income	11.9%	55.7%	91.2%	84.5%	101.1%	72.6%	69.1%	-
Expense category								
Food expenditures	$4.49	$13.30	$24.56	$21.92	$27.20	$21.07	$18.76	44%
Sex work expenditures	$1.04	$7.43	$11.65	$9.55	$13.33	$8.30	$8.55	20%
Housing expenditures	$0.46	$2.77	$6.23	$5.39	$6.40	$6.39	$4.61	11%
Other living expenditures ^c^	$1.13	$6.09	$7.05	$4.24	$6.13	$3.92	$4.76	11%
Education expenditures	$0.41	$2.79	$2.41	$7.99	$8.48	$3.61	$4.28	10%
Health care expenditures	$0.22	$1.46	$3.82	$3.12	$2.27	$1.67	$2.09	5%
Number of women with negative cash balance ^d^	7	30	49	48	67	54	120	-
Mean negative cash balance ^e^	−$94.80	−$88.75	−$94.60	−$81.37	−$72.79	−$56.07	−$81.40	-

^a^ Sum of individual income as reported in monthly financial diaries and computer-assisted surveys; ^b^ mean of the monthly means over the 6-month data collection period; ^c^ includes miscellaneous expenses, such as toiletries, leisure, cookware, etc.; ^d^ defined as cash expenditures > cash income; ^e^ average negative cash balance among women with a negative cash balance for the given month.

**Table 4:** Distribution of cash expenditure scenarios and HIV-related behaviors for women employed by sex (WESW) who were enrolled in the financial diaries sub-study (N = 163).

	N	(n/N) %
Cash expenditure scenario		
Had a negative cash balance ^a^ Yes No	120 43	73.6% 26.4%
Had high sex work costs ^b^ Yes No	46 117	28.2% 71.8%
Had high health care costs ^b^ Yes No	39 124	23.9% 76.1%
Had high education costs ^b^ Yes No	45 118	27.6% 72.4%
HIV-related behaviors		
Had ≥1 act(s) of condomless sex ^c^ Yes No	126 37	77.3% 22.7%
Had ≥1 acts of sex with alcohol/drugs ^c^ Yes No	114 49	69.9% 30.1%
Initiated HIV antiretroviral medication (ART/PrEP) Yes No	74 89	45.4% 54.6%

^a^ Defined as cash expenditures > cash income in any given month during 6-month financial diaries sub-study; ^b^ defined as WESW with average monthly costs over 6-month data collection period that are higher than 6-month average monthly costs for total sub-study sample (n = 163); calculated for sex work, health care, and education costs; ^c^ based on two most recent sexual partners in last 30 days.

**Table 5:** Proportion of women employed by sex work reporting sexual risk behaviors or uptake of antiretroviral medications (ART/PrEP) by cash expenditure scenario from financial diaries sub-study (N = 163).

	Negative Cash Balance		High Sex Work Costs		High Health Care Costs		High Education Costs	
	Yes	No	Yes	No	Yes	No	Yes	No
Total (N)	120	43	46	117	39	124	45	118
Condomless sex No Yes	29 (24.2%) 91 (75.8%)	8 (18.6%) 35 (81.4%)	11 (23.9%) 35 (76.1%)	26 (22.2%) 91 (77.8%)	8 (20.5%) 31 (79.5%)	29 (23.4%) 95 (76.6%)	12 (26.7%) 33 (73.3%)	25 (21.2%) 93 (78.8%)
Sex with alcohol or drugs No Yes	37 (30.8%) 83 (69.2%)	12 (27.9%) 31 (72.1%)	16 (34.8%) 30 (65.2%)	33 (28.2%) 84 (71.8%)	13 (33.3%) 26 (66.7%)	36 (29.0%) 88 (71.0%)	13 (28.9%) 32 (71.1%)	36 (30.5%) 82 (69.5%)
Initiated ART/PrEP No Yes	68 (56.7%) 52 (43.3%)	21 (48.8%) 22 (51.2%)	26 (56.5%) 20 (43.5%)	63 (53.8%) 54 (46.2%)	22 (56.4%) 17 (43.6%)	67 (54.0%) 57 (46.0%)	25 (55.6%) 20 (44.4%)	64 (54.2%) 54 (45.8%)

**Table 6:** Crude and adjusted odds ratios (OR) of association of sexual risk behaviors or uptake of antiretroviral medications by cash expenditure scenario (e.g., negative cash balance, high costs) among women employed by sex work who were enrolled in the financial diaries sub-study (N = 163).

	Negative Cash Balance		High Sex Work Costs		High Health Care Costs		High Education Costs	
	Crude OR (95% CI)	Adjusted OR ^a^ (95% CI)	Crude OR (95% CI)	Adjusted OR ^a^ (95% CI)	Crude OR (95% CI)	Adjusted OR ^a^ (95% CI)	Crude OR (95% CI)	Adjusted OR ^a^ (95% CI)
Condomless sex No Yes	1.00 0.72 (0.30, 1.72)	1.00 0.70 (0.28, 1.70)	1.00 0.91 (0.41, 2.03)	1.00 0.86 (0.38, 1.96)	1.00 1.18 (0.49, 2.86)	1.00 1.07 (0.43, 2.62)	1.00 0.74 (0.33, 1.64)	1.00 0.73 (0.32, 1.63)
Sex with alcohol or drugs No Yes	1.00 0.87 (0.40, 1.88)	1.00 0.93 (0.42, 2.05)	1.00 0.74 (0.36, 1.53)	1.00 0.76 (0.37, 1.59)	1.00 0.82 (0.38, 1.77)	1.00 0.85 (0.39, 1.86)	1.00 1.08 (0.51, 2.30)	1.00 1.04 (0.48, 2.22)
Initiated ART/PrEP No Yes	1.00 0.73 (0.25, 1.47)	1.00 0.80 (0.39, 1.67)	1.00 0.90 (0.45, 1.78)	1.00 0.99 (0.49, 2.03)	1.00 0.91 (0.44, 1.87)	1.00 1.10 (0.52, 2.35)	1.00 0.95 (0.48, 1.89)	1.00 0.90 (0.44, 1.85)

^a^ Adjusted for age and education status (less than primary education, primary school or more); * *p* < 0.05.

## Data Availability

Restrictions apply to the availability of these data due to the sensitive nature of the research. Requests may be made to the principal investigator, F.M.S.

## Abbreviations

ART	Antiretroviral therapy
AOR	Adjusted odds ratio
CI	Confidence interval
ESI	Economic-strengthening interventions
FD	Financial diary
GNI	Gross National Income
HIV	Human Immunodeficiency Virus
OR	Odds ratio
PrEP	Pre-exposure prophylaxis
SD	Standard deviation
UGX	Ugandan Shilling
USD	United States Dollar
WESW	Women employed by sex work
